# Treatment of Diphtheria

**Published:** 1863-02

**Authors:** 


### EDITORIAL DEPARTMENT.

### TREATMENT OF DIPHTHERIA.

How do you treat diphtheria? or how is diphtheria treated in Buffalo? is the daily inquiry of our correspondents, and for the purpose of answering to the best of our ability these questions from our friends, we will publish what we should otherwise very often write. We shall hardly attempt to answer fully the latter inquiry, but we can more easily, and perhaps more properly, speak of what we do ourselves in the treatment of this, to us, somewhat new, and important form of disease.

The *treatment* is the thing mostly inquired about; origin, nature, mode of propagation, diagnosis, natural history, prognosis, complications, &c., &c.

are fortunately for us, sufficiently well understood, and we have only to answer the inquiries, upon the subject of treatment. It may not however be inappropriate for us to say, before writing our prescriptions, that we regard the disease as a general or constitutional one, and benefitted mainly and almost exclusively by remedies addressed to the system generally, rather than by applications to the throat and fauces, which may or may not, manifest the usual exudation.

We are induced to consider this point a little, since it is not infrequent that we are asked, What applications do you make to the throat and fauces, or regard most valuable in the treatment of diphtheria? In some cases where the exudation was very abundant and extended into the larynx and upper portion of the trachea, constituting what is often called diphtheritic croup, we have been seduced into the use of the various topical remedies recommended. The severest case of this character we have seen recover, received no local applications whatever, and if we may be allowed to express our own private views upon a point where so great differences of opinion are entertained, we should give it as our growing conviction, that all applications made locally to the exudation when seen upon the tonsils and fauces, are not only worthless and distressing, but positively injurious, and that if the exudation is removed, nothing whatever is thereby gained towards overcoming the disease or mitigating the distress.

If we were brought to the confession, we should be forced to acknowledge, that previously, we did use local remedies, while at the same time, we should claim in mitigation that we were early in abandoning all caustic and irritating applications and advocating the more rational practice of local, "*non interference.*" We have seen the exudation often extend to the nasal passages and form in great abundance, and yet the amount, or extent of surface covered in no degree indicate the severity of the disease or its removal or disappearance mitigate the severity of the general symptoms; the prostration, frequent and feeble pulse, anorexia, oppressed and rapid respiration, cough, &c., &c. would yet remain unchanged. When by any alteration in the disease the exudation shall disappear, it does not constitute a cure, but simply the subsidence of one of the prominent symptoms of the disease, indicating a favorable termination, but by no means constituting such a result. It may be said, that the applications to the throat are not designed to effect the exudation only, but to overcome the irritation and inflammation of the mucous membrane. This is all very well, being sure that the irritation is not increased rather than diminished, and bearing in mind the limited surface which can be subjected to local medication.

The severer attacks of diphtheria are often fatal, while the disease in its milder and much more common form almost always terminates in recovery; that these terminations are greatly influenced by medication is by no means established, unless we make exception in favor of unfavorable result, since much may be done which is prejudicial, while less remains to be accomplished which is certainly beneficial. The natural history of this disease has not been, so far as we are informed, carefully noted; most cases have been subjected to some form of medication, and favorable results attributed to its influence.

After observing quite an extensive variety of cases in all forms of severity, and also noticing cases with, and without medication, we should now, if left to follow our own course, unbiased by any influences, recommend very little, and in some instances not any medicine. We should advise stimulants, food, beef essence, milk punch, and support generally, in any available form. Restlessness and pain we should allay with anodynes. We generally prescribe Dover's powder and quinine, but we have never been able to see any differences if the quinine was omitted. This article of the materia medica has grown into so universal favor that, whatever may be the disease, it is quite unsafe to neglect it; if the practitioner would leave nothing important for council to suggest, he must never omit quinine. When it is given in usual quantities, in conditions of debility, none of the common and visible effects of this remedy appear, and the only evidence of the action of the article or of its value, is to be found in subsequent improvement in the symptoms of the disease, which often appear, wholly independent of its influence. It is advised then in this disease upon theoretical belief in its tonic properties, rather than from any practical evidence of its value; it is given in this, as in almost all low forms of disease on account of its reputation as a tonic, while this reputation has been gained by its acknowledged and eminent virtues in controlling and arresting intermitting disease; its virtues as a tonic being much less fully established.

Chlorate of potash is an exceedingly fashionable remedy, and we are in the habit of prescribing it, mostly however from the certainty, that at least it will do no harm. That it exerts a favorable influence upon the urinary secretion is not improbable. Solution of chlorinated soda as a mouth-wash or gargle, affects as pleasantly the throat and fauces as any remedy with which we are acquainted, especially in cases of great fetor of the breath does this remedy appear efficacious.

To answer the question, how is diphtheria treated in Buffalo? giving the bill of fare in items and quantities, would hardly be consistent with our purpose. We have little doubt but all the different articles and plans recommended upon the face of the globe have been more or less faithfully tried, and cases terminating favorably have been regarded by some as cured by the medication and by others as having recovered in "spite" of it.

Alterative doses of calomel are considered as indispensable by a few practitioners, and its neglect or omission regarded as a fatal mistake, though we do not think physicians generally, or to any great extent, adopt this practice.

Local applications are more or less in use. Nitrate of silver, muriatic acid, sulphate of zinc, muriated tinc iron, &c., yet the use of these articles, has of late, greatly lessened.

We shall not regard it necessary to speak of the various complications of this disease, or the treatment they require, and yet we desire to say in passing, that diphtheritic croup, in some cases, so closely resembles membraneous croup, that we have no means of differential diagnosis. It often complicates many of the eruptive fevers; it may appear simultaneously with them, and with other diseases. In all cases, however, the general plan of treatment remains unchanged, though the severity and danger of the illness is greatly augmented.

There are a great many questions which suggest themselves without being asked, and we should be glad to consider many of them in this connection, but must defer for future opportunity. If any of our friends desire to answer for themselves the questions which have been so repeatedly proposed to us, our pages are open for the publication. We have only attempted to speak briefly upon the point of *treatment*, and have omitted almost everything which we most desire to say upon the subject of diphtheria.

